# Efficacy and safety of Qi-Jing Hui-Xin Decoction in the treatment of coronary microvascular angina: study protocol for a randomized, controlled, multi-center clinical trial

**DOI:** 10.1186/s13063-021-05508-x

**Published:** 2021-08-21

**Authors:** Yuanlong Sun, Chengxin Huang, Li Huo, Ying Li, Jun Chen, Zixiu Zhang, Meijun Jia, Meixian Jiang, Xiaofen Ruan

**Affiliations:** 1 Shuguang Hospital of Shanghai University of Traditional Chinese Medicine, Shanghai, 201203 China; 2 Cardiovascular Department, Shuguang Hospital of Shanghai University of Traditional Chinese Medicine, No. 528 Zhangheng Road, Pudong New Area, Shanghai, 201203 China; 3 Cardiovascular Research Institute of Traditional Chinese Medicine, Shuguang Hospital of Shanghai University of Traditional Chinese Medicine, Shanghai, 201203 China; 4 Cardiovascular Department, Shanghai Jing’an District Chinese Medicine Hospital, Shanghai, 200071 China; 5 Cardiovascular Department, Shanghai Yangpu Hospital of TCM, Shanghai, 200090 China

**Keywords:** Microvascular angina, Randomized controlled trial, Qi-Jing Hui-Xin Decoction, Traditional Chinese medicine, MACE, SAQ

## Abstract

**Background:**

With the increased understanding of heart disease, microvascular angina (MVA) is receiving greater attention from clinicians. Studies have shown that patients with MVA have significantly higher major cardiovascular events and all-cause mortality than the control population, and the search for effective treatments is of great clinical importance. Both basic and clinical studies have shown that Qi-Jing Hui-Xin Decoction (QJHX) can relieve angina symptoms and improve clinical efficacy, but there is a lack of high-quality clinical studies to provide a research basis. This article introduces the evaluation protocol of QJHX for the adjunctive treatment of MVA.

**Methods/design:**

This is a prospective randomized controlled trial. The trial will enroll 150 patients with MVA. On the basis of Western drug treatment, patients will be randomized into two groups, and the experimental group will receive QJHX treatment for 12 weeks and follow-up at 24 week. The primary indicators are the clinical efficacy of angina pectoris and the evidence of traditional Chinese medicine (TCM) efficacy. Secondary indicators are the Seattle Angina Scale score, serum lipid levels, electrocardiogram, and echocardiogram diagnosis. Additional indicators are endothelial function and immunoinflammatory factors. Adverse events will be monitored throughout the trial.

**Discussion:**

Integrated traditional Chinese and Western medicine is commonly used for angina in China. This study will evaluate the clinical effectiveness and safety of adding QJHX based on standardized Western medications. The results of the trial will provide high-level clinical research-based evidence for the application of QJHX in MVA.

**Trial registration:**

Chinese Clinical Trial Registry ChiCTR1900027015. Registered on 28 October 2019.

**Supplementary Information:**

The online version contains supplementary material available at 10.1186/s13063-021-05508-x.

## Administrative information

Note: the numbers in curly brackets in this protocol refer to Additional file 1 SPIRIT checklist item numbers. The order of the items has been modified to group similar items (see http://www.equator-network.org/reporting-guidelines/spirit-2013-statement-defining-standard-protocol-items-for-clinical-trials/

**Table:** 

Title [1]	Efficacy and safety of Qi-Jing Hui-Xin Decoction in the treatment of coronary microvascular angina: Study protocol for a randomized, controlled, multi-center clinical trial
Trial registration {2a and 2b}.	Chinese Clinical Trial Registry, ID: ChiCTR1900027015. Registered on October 28, 2019.
Protocol version {3}	11 November 2018, version 2.0
Funding {4}	This study was funded by (1) Shanghai Shenkang development research project (SHDC12018X29); (2) Shanghai Promoting TCM 3-Year Action Program (ZY(2018-2020)-RCPY-2004); (3) National Natural Science Foundation of China (NO.81403352)
Author details {5a}	(1) Yuanlong Sun, Shuguang Hospital of Shanghai University of Traditional Chinese Medicine, Shanghai, China; sunyuanlong@shutcm.edu.cn; (2) Chengxin Huang, Cardiovascular Research Institute of Traditional Chinese Medicine, Shuguang Hospital of Shanghai University of Traditional Chinese Medicine, Shanghai, China; huangchengxin96@163.com (3) Li Huo, Cardiovascular Department, Shuguang Hospital of Shanghai University of Traditional Chinese Medicine, Shanghai, China; hl970204@163.com; (4) Ying Li, Cardiovascular Department, Shanghai Jing’an District Chinese Medicine Hospital, Shanghai, China; llyy018@163.com (5) Jun Chen, Cardiovascular Department, Shanghai Yangpu Hospital of TCM, Shanghai, China; chenjun72514@163.com (6) Zixiu Zhang, Cardiovascular Department, Shanghai Yangpu Hospital of TCM, Shanghai, China; zhangzixiu_12@126.com; (7) Meijun Jia, Cardiovascular Department, Shuguang Hospital of Shanghai University of Traditional Chinese Medicine, Shanghai, China; 1852jmj@shutcm.edu.cn (8) Meixian Jiang, Cardiovascular Department, Shuguang Hospital of Shanghai University of Traditional Chinese Medicine, Shanghai, China; jmx591@126.com; (9) Xiaofen Ruan, Cardiovascular Department, Shuguang Hospital of Shanghai University of Traditional Chinese Medicine, Shanghai, China; ruanxiaofeng@shutcm.edu.cn
Name and contact information for the trial sponsor {5b}	Sponsor: Shuguang Hospital of Shanghai University of Traditional Chinese Medicine, Zhangheng Road 528, Pudong New Area, Shanghai, China Coordinating Investigator (contact): Prof. Dr. Xiaofen Ruan Cardiovascular Department Shuguang Hospital of Shanghai University of Traditional Chinese Medicine Zhangheng Road 528 Pudong New Area, Shanghai, China Tel: 086-021-53827250 Email: ruanxiaofeng@shutcm.edu.cn;
Role of sponsor	The sponsor has no role in the design of the study, in the collection, analysis, and interpretation of the data, and writing of the manuscript

## Introduction

### Background

Microvascular angina (MVA) is a clinical syndrome manifested by exertional angina or myocardial ischemia caused by dysfunction of small anterior coronary arteries and small arteries [2]. Currently, the etiology of MVA is thought to be related to coronary microcirculation dysfunction (CMD), coronary endothelial dysfunction, low estrogen levels, and inflammatory response [3]. According to a survey, approximately 112 million people worldwide suffer from angina pectoris [4]. Nearly 70% of patients who underwent coronary angiography showed normal arteries but still had symptoms of myocardial ischemia, and there were more women than men [5]. In a Danish study of 11,223 patients with stable angina who underwent coronary angiography, 32% of men showed non-obstructive coronary artery disease (CAD), compared to 65% of women, and this proportion has been increasing [6]. According to the survey, CMD is the most common type of MVA, and some patients also present with the mixed epicardial and microvascular type of vasospasm caused by epicardial spasm [7]. Currently, statins, angiotensin-converting enzyme inhibitors (ACEIs), and calcium antagonists are the main drugs for the treatment and relief of MVA [2]. However, the results of these drugs have not been uniform in different clinical studies, leading to recurrent disease and poor quality of life in patients [8]. In addition, Western medicine alone is ineffective, and multiple drugs have significant side effects. There is still a need to develop more effective treatments for MVA. In China, a study has confirmed that the combination of Chinese and Western medicines is significantly better than the use of Western medicines alone for the treatment of MVA [9].

According to its manifestations and clinical characteristics, MVA is called “chest pain,” “heartache,” and “true heartache” in traditional Chinese medicine (TCM). Zhang believes that the pathogenesis of thoracic obstruction is the deficiency in origin and excess in superficiality; thus, the reinforcing method is one of the effective methods to treat MVA [10]. Qi-Jing Hui-Xin Decoction (QJHX), a formula for the treatment of angina pectoris, consists of 10 herbs, including *Astragalus propinquus*, *Polygonatum sibiricum*, *Ziziphi spinosae*, and *Panax notoginseng*. The positive clinical efficacy of QJHX for coronary heart disease has been shown in our previous studies that QJHX can reduce angina scores in patients with CAD compared to Western medicine treatment alone. Meanwhile, experimental studies have also confirmed that QJHX can improve cardiac function in rats with myocardial ischemia. In this study, we propose to perform a multicenter randomized controlled clinical study to further observe the clinical efficacy of QJHX intervention in MVA patients and its effect on long-term prognosis.

## Methods/design

### Study design

**Fig. 1:**
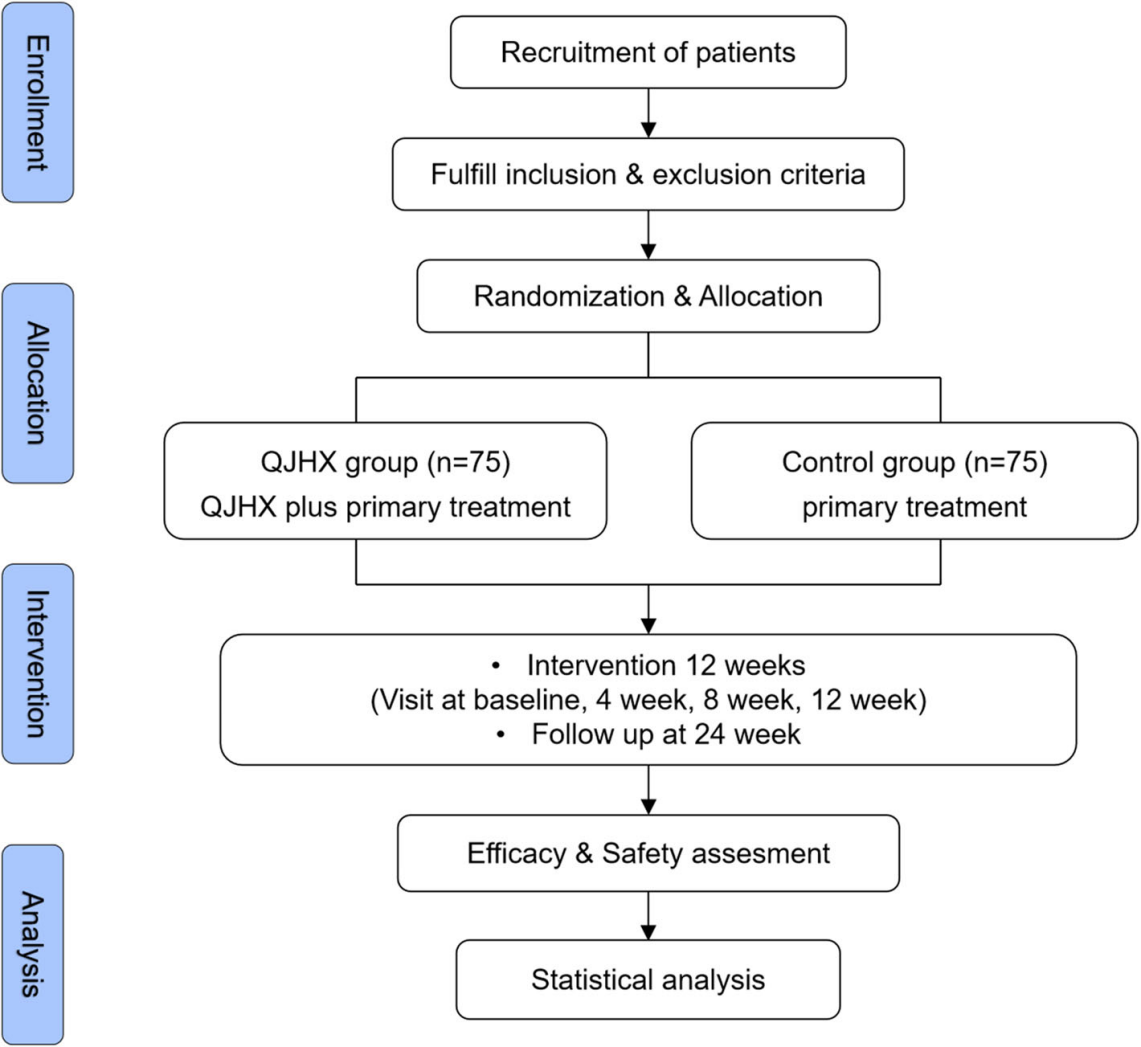
Flow diagram of this study protocol

This study is a multi-center, prospective, randomized controlled trial designed to evaluate the efficacy and safety of QJHX in patients with coronary MVA based on standardized Western treatment. A flow diagram of this study protocol is shown in Fig. 1.

**Fig. 2:**
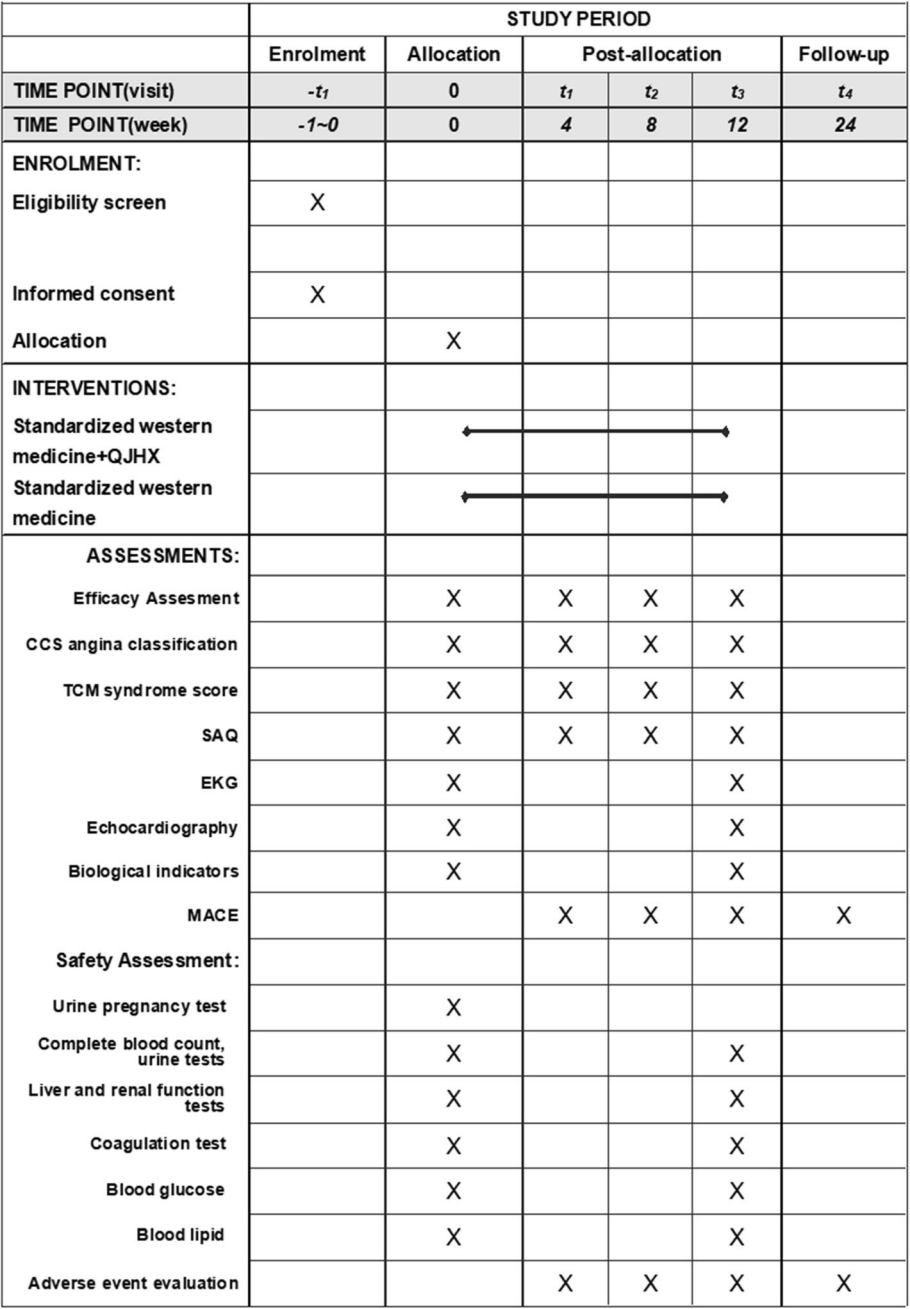
Study schedule of enrolment, interventions, and assessments. QJHX, Qi-Jing Hui-Xin Decoction; CCS, Canadian Cardiovascular Society; TCM, traditional Chinese medicine; SAQ, Seattle angina questionnaire; EKG, electrocardiogram; MACE, major adverse cardiac events

To avoid regional differences, the study will be conducted in three hospitals from different areas of Shanghai. The points of data collection in this trial are as follows: intervention period (12 weeks): baseline, medication for 4 weeks ±3 days, medication for 8 weeks ±3 days, medication for 12 weeks ±3 days for hospital visits, and telephone follow-up for 12 weeks ±7 days after the end of the intervention. The corresponding items will be measured according to the time point of data collection. The detailed information is shown in Fig. 2.

### Sample size

According to the results of a previous single-center clinical observation, after 12 weeks of treatment with QJHX, the effective rate was 85.7% in the treatment group and 60.1% in the control group. According to the sample calculation decoction, the number of samples is *n* = 61 cases in each group. In the decoction, *p*_*1*_ and *p*_*2*_ are the estimated values of the two population rates, respectively, *p* is the combined rate, *p* = (*p*_*1*_ + *p*_*2*_)/2, when *α* = 0.05, *β* = 0.10, *u*_α_ = 1.96, and *u*_β_ = 1.282 in the double test and bilateral test. The sample size was 75 cases in each group, a total of 150 cases, taking into account a 20% drop-off rate.

$$ {\mathrm{n}}_1={n}_2{\frac{\left[ ua\sqrt{2p\left(1-p\right)}+ u\beta \sqrt{p_1\left(1-{p}_1\right)+{p}_2\left(1-{p}_2\right)}\right]}{{\left({p}_1-{p}_2\right)}^2}}^2 $$

### Eligibility criteria

Eligible patients will be those who meet all of the following inclusion criteria and do not have any of the listed exclusion criteria.

### Diagnostic criteria

1. The diagnostic criteria of primary stable MVA [2, 11]: (1) typical exertional angina symptoms, but nitroglycerin is ineffective. (2) Objective evidence of myocardial ischemia (ST-segment depression, myocardial perfusion defect, or increased myocardial metabolites) under resting or loading conditions, but no segmental ventricular wall motion abnormality. (3) The case fatality ratio (CFR) measured by noninvasive or traumatic imaging technology is < 2.0. (4) There was no obvious subepicardial coronary artery stenosis (< 20%) by coronary angiography or coronary computed tomography. (5) Non-cardiogenic chest pain and other heart diseases are excluded.
2. Microvascular angina pectoris with obstructive CAD: (1) in patients with stable angina pectoris caused by obstructive CAD, such as long-term angina pectoris, severe angina pectoris, and angina pectoris that is readily induced by physical activity, sublingual nitroglycerin is ineffective. (2) The CFR measured by noninvasive or traumatic imaging technology is < 2.0.
3. The angina pectoris severity diagnostic criteria are classified by following the “American College of Cardiology (ACC)/American Heart Association (AHA)/American College of Physicians (ACP-ASIM) Chronic Stable Angina Pectoris Management Guidelines” and “Canadian Cardiovascular Society (CCS) Angina Pectoris Severity Classification.”

### Inclusion criteria

1. Age over 18 years
2. Patients conform to the diagnostic criteria of MVA (CSS angina severity grades I–III)
3. TCM syndrome meet the Guideline for Clinical Study on Angina Pectoris of CHD Treated with Traditional Chinese Medicines and Natural Medicines [12]
4. Sign informed consent and willingness to accept Chinese medicine treatment

### Exclusion criteria

1. Severe heart disease (unstable angina, severe arrhythmia, and others), chest pain caused by diseases other than CAD, such as psychosis, severe neurosis, hyperthyroidism, biliary heart syndrome, gastroesophageal reflux, and aortic dissection
2. Complicated with uncontrolled grade III hypertension (systolic blood pressure (> 180 mmHg) and/or diastolic blood pressure (> 110 mmHg), severe cardiopulmonary dysfunction, and severe arrhythmia
3. The patients with liver, kidney, hematopoietic system, and other serious diseases
4. Those who have participated in other drug clinical trials within 1 month
5. Those who have recently (over 4 weeks) undergone major surgery
6. Pregnancy, lactating women, or pregnant planners
7. Anaphylaxis or allergic reaction to the test drug ingredients

### Randomization and concealment

Eligible patients will be randomly assigned to the treatment group or the control group in a 1:1 ratio, using SAS® 9.4 statistical software and a site-stratified, block randomization schedule. Randomized allocation sequence of the trial participants will be sealed in opaque envelopes and kept in a double-locked cabinet in an independent data center. Each participating hospital will have an independent drug administrator responsible for random coding and drug management.

### Recruitment

The investigators (YS, CH, LH, and MJ at Shuguang Hospital; YL at Jing’an District Chinese Medicine Hospital; and JC and ZZ at Yangpu Hospital) will be trained beforehand and provided with a printed standardized protocol, informed consent form, and case report form (CRF). Patients will be informed of the probable benefits and potential risks, and they will be assured that participation is entirely voluntary. Patient enrollment will be performed after providing signed informed consent.

The investigators (YS, LH, CH, MJ, YL, JC, and ZZ) will fill out the CRF with participant data and collect the biological specimens from participants after they have been informed of the trial process. The personal information of all participants will always be kept confidential. Any argument about recruitment between investigators will be accessed and determined by professor MJ and XR.

Participants will be free to withdraw at any time during the trial. Participants who wish to withdraw will be offered the option to cease trial medication but continue attending scheduled visits for outcome measurement. Participants who withdraw will be followed up to investigate the reason for withdrawal. Participants may be advised to discontinue the treatment if there is a product-related adverse event of a serious nature or if the participant was not compliant with the study requirements. Discontinuers will not be replaced by new participants. Intention-to-treat analysis will be performed on missing data from discontinuers with the last observation carried forward method. If moderate or severe adverse reactions judged to be related to the clinical trial occur in more than 25% of the total participants, or if the required number of participants has not been fully enrolled within the schedule, this clinical trial may be terminated early.

### Interventions

Eligible participants will be randomized to either the test group or the control group. According to the patient’s condition, the following primary treatments will be selected: antiplatelet drugs (such as aspirin), statins, β-blockers, calcium antagonists, and nitrates. Participants in the control group will receive primary treatment, while QJHX plus primary treatment will be applied in the test group.

The TCM decoction is a compound preparation of Chinese herbs, whose ingredients are equivalent to *Astragalus propinquus*, *Polygonatum sibiricum*, *Ziziphi spinosae*, and *Panax notoginseng*; 10 herbals in total, packaged in a unit of 3 g. QJHX will be produced by Jiangyin Tianjiang Pharm Co. Ltd. (approval number AH20160363), Jiangsu, China. Drugs will be packaged and distributed according to the random serial number in the drug distribution center.

The dosage, method, and frequency of administration will be explained to the participants orally and in writing. Participants will be instructed to take one pack (3.0 g) at a time, two times per day for 12 weeks. At each visit, the participants will receive the drugs for the next intervention, and the drugs left over from the previous intervention will be returned. The dose taken and the accordingly calculated medication compliance will be recorded in the CRF. The scheduled treatment time will be 12 weeks unless serious adverse events occur or the patient withdraws from treatment. During the observation period, the basic treatment of coronary heart disease is unchanged, and non-pharmacologic care is permitted. Other Chinese herbal medicines, Chinese patent medicines, and psychiatric medication are prohibited. If angina pectoris occurs, nitroglycerin tablets can be temporarily taken. Any combination of medications and doses for physical conditions will be recorded in detail. Any questions raised by participants will be answered to facilitate the completion of the trial. The detailed study schedule is shown in Fig. 2.

### Outcomes

The primary outcome is Angina Scale and TCM syndrome score according to the guiding principles of clinical research technology of traditional Chinese medicine and natural medicine in the treatment of coronary heart disease and angina pectoris. The higher the score, the more severe angina pectoris and symptoms. The secondary outcomes are based on the Seattle Angina Questionnaire (SAQ), another scale of angina; three scales will be collected at baseline, 4, 8, and 12 weeks after randomization. Serum total cholesterol [13], triglyceride [14], high-density lipoprotein cholesterol (HDL-C), low-density lipoprotein cholesterol (LDL-C), non-high-density lipoprotein cholesterol (non-HDL-C), apolipoprotein [8] A-I, ApoA-II, ApoB, lipoprotein-a, and the proportions of related biochemical parameters, including TC/HDL-C, non-HDL-C/HDL-C, and ApoB/Apo A-1, endothelin (ET), nitric oxide (NO), prostaglandin E (PGE), calcitonin gene-related peptide (CGRP), hypersensitive-C-reactive-protein (hs-CRP), and tumor necrosis factor α (TNF-α); the results of transthoracic Doppler echocardiography (TTDE), including left ventricular end-diastolic diameter (LVEDD), left ventricular end-systolic diameter (LVESD), left atrium (LA), interventricular septal thickness (IVST), left ventricular posterior wall (LVPW), fractional shortening (FS), and left ventricular ejection fraction (LVEF); an electrocardiogram (EKG) will be measured and collected at baseline and 12 weeks after randomization.

Safety outcomes are blood routine examination and laboratory liver and renal function test results, including aspartate aminotransferase (AST), alanine aminotransferase (ALT), alkaline phosphatase (ALP), blood urea nitrogen (BUN), creatinine (CR), and uric acid (UA). Adverse events will be monitored at each visit. The investigators will evaluate the degree of adverse events and whether they are related to the intervention, and they will ensure that the adverse events are properly resolved. All collected adverse events and subsequent medical intervention will be recorded in the CRF.

### Data management

Data management and statistical analysis will be performed with an electronic data capture system (EDC) by the statistical department in Shuguang hospital. All personal information and biochemical and physicochemical results about the potential and enrolled participants will be collected and kept by the doctors in charge to protect confidentiality.

An independent clinical research associate [15] will regularly audit and monitor the study at each hospital. Any protocol revisions will be reviewed and approved by the ethics committee of Shuguang Hospital. Any modifications to the protocol will be reapproved by the Ethics Committee of Shuguang Hospital. The results of this trial will be submitted for publication in peer-reviewed journals and can be disseminated through conference presentations.

### Statistical analysis

Continuous variables are expressed as mean ± standard deviation [16] or median (quartile range), depending on the nature of the distribution, and they will pass two-sample *t* tests (assuming normal distribution and equal variance), separate-variance *t* test (without assuming normal distribution and mean squared error), or the Mann-Whitney *U* test (without assuming normal distribution and mean squared error). Categorical variables are expressed as frequency and percentage, and they will be compared between groups by the chi-square test or Fisher’s exact test. Statistical analysis will be performed using SPSS 22.0 software.

## Discussion

This is the study protocol for an investigator-initiated preliminary clinical trial evaluating the effects of QJHX in patients with MVA. We will evaluate the efficacy and safety of this decoction compared to a control group and assess the feasibility of a large-scale randomized controlled trial. First, we will screen the patients for diagnosis based on the diagnostic criteria for coronary microvascular lesions. After that, the patients will be graded and scored with confirmed MVA according to the angina grading criteria. At the same time, the patients will be diagnostically staged and scored according to the TCM clinical research documents. Finally, we will develop the inclusion criteria, exclusion criteria, and randomly grouped subjects who met the inclusion criteria.

QJHX is mainly used to treat CAD by the reinforcing method. We will identify the subjects in the experimental group according to the TCM pattern identification and typing criteria and administer the medicine according to the dosing method. In addition, to evaluate the safety of the drugs, we will perform routine blood tests and liver and kidney function tests at baseline and after 12 weeks of treatment.

Herbal medicines provide the advantage of multiple active ingredients acting synergistically on multiple targets for disease treatment compared to single active ingredients of Western medicines [17]. Recent studies have shown that CMD is a major cause of MVA and is one of the main influencing factors in the development of MVA. Early detection of CMD can help predict the occurrence of MVA and is expected to intervene early in stopping disease progression [18]. Furthermore, coronary artery endothelial dysfunction and inflammatory response are jointly involved in the development of CMD [19]. Several herbs in QJHX have been experimentally proven to be effective against CMD. Astragalus polysaccharide (APS), one of the main bioactive components of Astragalus, plays a protective role against cardiac vascular endothelial dysfunction in rats through anti-inflammation and improvement in the imbalance between reactive oxygen species and NO [20]. Another component of Astragalus, Astragaloside IV (AGIV), can improve vascular endothelial dysfunction through the TLR4/NF-κB signaling pathway [21]. In addition, AGIV promotes the release of eNOS via the PI3K/Akt/eNOS signaling pathway, which induces a vasodilatory response [22]. Meanwhile, the other main herb of this formula, *Polygonatum*, which contains *Polygonatum sibiricum* polysaccharides (PSP), has anti-inflammatory and anti-oxidative stress effects [1, 23], which may prevent the development of CMD by inhibiting the occurrence of an inflammatory response. In addition, some studies have shown that PSP can also protect endothelial cells [24]. *Panax notoginseng* Saponins, the main component of Panax ginseng, inhibits intimal hyperplasia in rat arteries by suppressing the pERK/p38mapk pathway [25]. In addition, Wenxin Keli with *Polygonatum* and *Panax notoginseng* as the main ingredients can significantly improve myocardial ischemia and relieve angina pectoris in coronary heart disease [26, 27].

In conclusion, this paper presents a rigorously designed, randomized controlled clinical study protocol for the treatment of MVA with QJHX. In this study, we will observe the changes in safety indicators and efficacy indicators, such as angina pectoris score, serum lipids, ECG, cardiac ultrasound, TCM evidence score, and quality of life scale; vascular endothelial function indicators and immune and inflammatory factors; and the number of outpatient visits and hospitalizations due to symptom exacerbation by telephone follow-up after 6 months. The results of this study will provide a basis for a large-scale randomized controlled study to further evaluate the efficacy and safety of QJHX in the treatment of primary microvascular angina pectoris, to preliminarily elucidate the mechanism of action, and to accumulate information for preclinical studies of new drugs.

## Trial status

The final protocol version is 2.0 and is dated 11 November 2018. Recruitment began on 14 November 2019, and it will be completed on 23 November 2021. This study has recruited 83 patients so far.

## Supplementary Information

**Additional file 1.**

## Data Availability

The datasets used and/or analyzed during the current study are available from the corresponding author on reasonable request.

## Abbreviations

MVA: Microvascular angina
QJHX: Qi-Jing Hui-Xin Decoction
CCS: Canadian Cardiovascular Society
TCM: Traditional Chinese medicine
SAQ: Seattle angina questionnaire
EKG: Electrocardiogram
MACE: Major adverse cardiac events
CMD: Coronary microcirculation dysfunction
CAD: Coronary artery disease
CFR: Case fatality ratio
CRF: Case report form
TTDE: Transthoracic Doppler echocardiography
CRA: Clinical research associate
SD: Standard deviation
APS: Astragalus polysaccharide
AGIV: Astragaloside IV
PSP: *Polygonatum sibiricum* polysaccharides
